# A clioquinol-containing Pluronic^®^ F127 polymeric micelle system is effective in the treatment of visceral leishmaniasis in a murine model

**DOI:** 10.1051/parasite/2020027

**Published:** 2020-04-30

**Authors:** Grasiele S.V. Tavares, Débora V.C. Mendonça, Isabela A.G. Pereira, João A. Oliveira-da-Silva, Fernanda F. Ramos, Daniela P. Lage, Amanda S. Machado, Lívia M. Carvalho, Thiago A.R. Reis, Luísa Perin, Ana Maria R.S. Carvalho, Flaviano M. Ottoni, Fernanda Ludolf, Camila S. Freitas, Raquel S. Bandeira, Alessandra M. Silva, Miguel A. Chávez-Fumagalli, Mariana C. Duarte, Daniel Menezes-Souza, Ricardo J. Alves, Bruno M. Roatt, Eduardo A.F. Coelho

**Affiliations:** 1 Medicina, Universidade Federal de Minas Gerais Belo Horizonte Minas Gerais Brazil; 2 Departamento de Produtos Farmacêuticos, Faculdade de Farmácia, Universidade Federal de Minas Gerais 31270-901 Belo Horizonte Minas Gerais Brazil; 3 Laboratório de Imunopatologia, Núcleo de Pesquisas em Ciências Biológicas/NUPEB, Departamento de Ciências Biológicas, Insituto de Ciências Exatas e Biológicas, Universidade Federal de Ouro Preto Ouro Preto Minas Gerais Brazil; 4 Universidad Católica de Santa María, Urb. San José S/N Umacollo Arequipa Peru; 5 Departamento de Patologia Clínica, COLTEC, Universidade Federal de Minas Gerais Belo Horizonte Minas Gerais Brazil

**Keywords:** Treatment, Visceral leishmaniasis, Clioquinol, Immune response, Delivery systems, Miltefosine

## Abstract

A clioquinol (ICHQ)-containing Pluronic^®^ F127 polymeric micelle system (ICHQ/Mic) was recently shown to be effective against *Leishmania amazonensis* infection in a murine model. In the present study, ICHQ/Mic was tested against *L. infantum* infection. BALB/c mice (*n* = 12 per group) were infected with *L. infantum* stationary promastigotes through subcutaneous injection and, 45 days after challenge, received saline or were treated via the subcutaneous route with empty micelles, ICHQ or ICHQ/Mic. In addition, animals were treated with miltefosine by the oral route, as a drug control. Half of the animals were euthanized 1 and 15 days after treatment, aiming to evaluate two endpoints after therapy, when parasitological and immunological parameters were investigated. Results showed that the treatment using miltefosine, ICHQ or ICHQ/Mic induced significantly higher anti-parasite IFN-γ, IL-12, GM-CSF, nitrite and IgG2a isotype antibody levels, which were associated with low IL-4 and IL-10 production. In addition, a higher frequency of IFN-γ and TNF-α-producing CD4^+^ and CD8^+^ T-cells was found in these animals. The parasite load was evaluated in distinct organs, and results showed that the treatment using miltefosine, ICHQ or ICHQ/Mic induced significant reductions in organic parasitism in the treated and infected mice. A comparison between the treatments suggested that ICHQ/Mic was the most effective in inducing a highly polarized Th1-type response, as well as reducing the parasite load in significant levels in the treated and infected animals. Data obtained 15 days after treatment suggested maintenance of the immunological and parasitological responses. In conclusion, ICHQ/Mic could be considered in future studies for the treatment of visceral leishmaniasis.

## Abstract (fr)

Un système à micelles polymériques Pluronic^®^ F127 (ICHQ/Mic) contenant du clioquinol (ICHQ) s’est récemment révélé efficace contre l’infection à *Leishmania amazonensis* dans un modèle murin. Dans la présente étude, l’ICHQ/Mic a été testé contre l’infection à *L. infantum*. Les souris BALB/c (*n* = 12 par groupe) ont été infectées par des promastigotes stationnaires de *L. infantum* par injection sous-cutanée et ont reçu 45 jours après l’épreuve une solution saline ou ont été traitées par voie sous-cutanée avec des micelles vides, ICHQ ou ICHQ/Mic. De plus, les animaux ont été traités avec de la miltefosine par voie orale, comme contrôle médicamenteux. La moitié des animaux ont été euthanasiés 1 et 15 jours après le traitement, dans le but de mesurer deux critères d’évaluation après la thérapie, lorsque les paramètres parasitologiques et immunologiques ont été étudiés. Les résultats ont montré que le traitement par miltefosine, ICHQ ou ICHQ/Mic induisait des niveaux d’anticorps anti-parasite IFN-γ, IL-12, GM-CSF, nitrite et IgG2a significativement plus élevés, associés à de faibles productions d’IL-4 et IL-10. De plus, une fréquence plus élevée de cellules T CD4^+^ et CD8^+^ produisant de l’IFN-γ and TNF-α a été trouvée chez ces animaux. La charge parasitaire a été évaluée dans des organes distincts et les résultats ont montré que le traitement utilisant la miltefosine, ICHQ ou ICHQ/Mic induisait des réductions significatives du parasitisme des organes chez les souris traitées et infectées. Une comparaison entre les traitements a suggéré qu’ICHQ/Mic était le plus efficace pour induire une réponse de type Th1 polarisée, ainsi que pour réduire la charge parasitaire à des niveaux significatifs chez les animaux traités et infectés. Les données obtenues 15 jours après le traitement suggèrent le maintien des réponses immunologiques et parasitologiques. En conclusion, ICHQ/Mic pourrait être envisagé dans de futures études pour le traitement contre la leishmaniose viscérale.

## Introduction

Leishmaniases are diseases caused by parasitic protozoa belonging to more than 20 different *Leishmania* species [61]. Distinct clinical manifestations of this disease complex are found in infected mammalian hosts, ranging from self-curing cutaneous lesions to life-threatening visceral disease [60]. Visceral leishmaniasis (VL) is caused by *Leishmania donovani* species in Asia and Africa, and by *L. infantum* in the Mediterranean Basin, Middle East and the Americas. Acute disease, which is characterized by several symptoms, such as fever, anemia, weight loss and fatigue, can be fatal if left untreated [12, 28]. About 0.2–0.4 million VL cases occur each year, of which the majority are reported in India, where the disease is an important public health problem [52]. In the Americas, Brazil accounts for about 90% of the VL cases recorded annually [60].

Since it is often difficult to rapidly and precisely diagnose VL, and no human vaccines are available, treatment of VL should be improved. However, there are problems associated with the side effects caused by drugs, besides the prolonged hospitalization time, high cost, and/or the emergence of parasite resistance [20, 54]. Amphotericin B (AmpB) is a known antifungal agent that has shown effective antileishmanial activity against distinct *Leishmania* species [5, 43, 45]. The mechanism of action of the drug was related to binding to ergosterol present in the parasite membrane, hampering cell permeability, and causing the loss of cations and cell death [9]. However, the use of AmpB has been limited, mainly due to drug toxicity, which can cause nephrotoxicity, cardiac changes, hemolysis, and liver damage [50].

AmpB-based liposomal formulations are better tolerated than the free drug. They also present a high therapeutic index, short treatment period, and higher safety for patients [23]. However, these formulations have high cost and, as a consequence, their use is limited [39]. AmBisome^®^ is an AmpB-based liposome formulation used as first-line therapy against VL, and in some countries, such as India, it is provided at a reduced cost and/or as a donation [62]. Miltefosine has also been used to treat VL, administered via the oral route. However, the drug is teratogenic and parasite resistance has been identified [16, 49]. In this context, there is a need to identify new antileishmanial compounds that are effective against different *Leishmania* species, and that have a low cost, with the aim of improving the quality of treatment.

Not less important, drug delivery systems have been used to circumvent the toxicity of older drugs, but without losing their biological effect [43]. These systems include nanoparticles, microspheres and micelles, which are applied to reduce the toxicity of conventional drugs, such as AmpB [1, 4, 25, 41]. Poloxamer 407^®^ is a thermo-reversible co-polymer with an amphiphilic nature, consisting of hydrophilic and hydrophobic segments. Such compounds are inexpensive and easily manufactured, and have good stability and efficient targeting ability [40]. In this context, Poloxamer 407-based micelles have been developed and evaluated as delivery systems to treat various diseases [59, 63, 31].

Recently, a flavonoid called 8-hydroxyquinoline (8-HQN) was incorporated into a Poloxamer 407-based system (8-HQN/M), and the composition was found to be effective in treating *L. amazonensis* infection in BALB/c mice [30]. In another study, clioquinol (5-chloro-7-iodoquinolin-8-ol or ICHQ), an 8-HQN derivate, also showed *in vitro* antileishmanial activity against *L. amazonensis* and *L. infantum* species [55], alongside *in vivo* activity against murine tegumentary leishmaniasis (TL) [56]. In this study, ICHQ was incorporated into Poloxamer 407 micelles (ICHQ/M), and the composition was evaluated in *L. amazonensis*-infected BALB/c mice versus the use of AmpB and AmBisome^®^. Results suggested that ICHQ and ICHQ/M-treated mice presented the highest reductions in their average lesion diameter and parasite burden in the infected tissue, spleen, liver and draining lymph nodes of the animals, which were correlated with the development of antileishmanial Th1-type response, based on production of IFN-γ, IL-12, TNF-α, and GM-CSF. No toxicity was found in the treated and infected animals [56].

With the aim of identifying new antileishmanial targets, in the present study, the ICHQ/Mic composition was evaluated against *L. infantum* infection. Miltefosine was used as a drug control. The compounds were administered by the subcutaneous or oral route (miltefosine) in the infected mice, and the efficacy of the treatments was evaluated one and 15 days after therapy, when parasitological and immunological parameters were investigated by specific techniques, such as a limiting dilution assay, quantitative real-time PCR (RT-PCR), capture ELISA, and flow cytometry.

## Materials and methods

### Mice and parasites

The study was approved by the Committee for the Ethical Handling of Research Animals of the Federal University of Minas Gerais (UFMG; Belo Horizonte, Minas Gerais, Brazil), under protocol number 085/2017. Female BALB/c mice (8 weeks old) were purchased from the Institute of Biological Sciences of UFMG, and were kept under pathogen-free conditions. The *L. infantum* (MHOM/BR/1970/BH46) strain was used. Stationary promastigotes were grown in Schneider’s medium (Sigma-Aldrich, USA) containing 20% heat-inactivated fetal bovine serum (FBS; Sigma-Aldrich, USA) and 20 mM L-glutamine at pH 7.4 in 24 °C [10].

### Preparation of the ICHQ-containing micelles

Poloxamer 407 (Pluronic^®^ F127), ICHQ, and miltefosine were purchased from Sigma-Aldrich (St. Louis, MO, USA), with catalog numbers 16758, 130-26-7, and 58066-85-6, respectively. ICHQ-containing micelles (ICHQ/M) were prepared as described [56]. Briefly, Poloxamer 407 (18% w/w) was diluted in phosphate buffer (PBS 1×) at pH 7.4 under magnetic agitation for 18 h and 4 °C. Eight milligrams of the molecule were added to 500 μL of dichloromethane and solubilized using vortex. The mixture was added to the previously prepared solution under vigorous magnetic agitation and in an ice bath until a viscous emulsion was obtained. The dichloromethane was evaporated using a rotary evaporator (Buchi, Flawil, Switzerland), and the ICHQ-containing composition was obtained as a transparent yellow gel at room temperature. Empty micelles were prepared (18% w/w) using the same protocol.

### Infection and treatment schedule

Mice (*n* = 12 per group) were infected with 10^7^ *L. infantum* stationary promastigotes through subcutaneous injection. Forty-five days post-infection, animals were divided into groups and received one of the following treatment schedules by the subcutaneous route, every 2 days for 10 days: (a) control (saline) group: mice received 50 μL of PBS 1× pH 7.4; (b) empty micelle (Mic/B) group: mice received 50 μL of micelles (10 mg/kg body weight); (c) miltefosine group: mice received 2 mg/kg body weight of drug, which was applied by the oral route; (d) ICHQ group: mice received 50 μL of ICHQ (10 mg/kg body weight); and (e) ICHQ/micelle (ICHQ/Mic) group: mice received 50 μL of ICHQ-containing micelles (5 mg/kg body weight). Half of the animals were euthanized one and 15 days after treatment, when parasitological and immunological parameters were evaluated.

### Parasite load evaluated by limiting dilution

The parasitism in the treated and infected mice was investigated in their spleen, liver, bone marrow (BM) and draining lymph nodes (dLN), one and 15 days after treatment, by using alimiting dilution assay [15]. Briefly, organs were macerated in a glass tissue grinder using sterile PBS, and tissue debris were removed by centrifugation at 150 ×*g*. Cells were concentrated by centrifugation at 2,000 ×*g*, pellets were resuspended in 1 mL of complete Schneider’s medium and a log-fold serial dilution was performed in Schneider’s medium (10^−1^–10^−12^ dilution). Each sample was plated in triplicate, and read 7 days after the beginning of the cultures, at 24 °C. Results were expressed as the negative log of the titer (the dilution corresponding to the last positive well) adjusted per milligram of organ.

### Splenic parasitism evaluated by RT-PCR

Splenic parasitism was also evaluated using an RT-PCR technique [17]. Briefly, spleen DNA was extracted using a Wizard Genomic DNA Purification Kit (Promega Corporation), according to the manufacturer’s recommendations. The DNA was resuspended in 100 μL of milli-Q water, and the parasite burden was estimated using the following primers to amplify the *L. infantum* kDNA: *Forward* (CCTATTTTACACCAACCCCCAGT) and *Reverse* (GGGTAGGGGCGTTCTGCGAAA). The Mouse β-actin gene (*Forward*: CAGAGCAAGAGAGGTATCC; *Reverse*: TCATTGTAGAAGGTGTGGTGC) was used as an endogenous control to normalize nucleated cells and single-copy-number, as well as to verify sample integrity. Standard curves were obtained from DNA extracted from 1 × 10^8^ parasites for kDNA and 1 × 10^8^ peritoneal macrophages for β-actin, under the same conditions used to extract the samples used in the present study. Reactions were processed and analyzed in an ABI Prism 7500 Sequence Detection System (96 well-plate; Applied Biosystems) using 2× SYBR^TM^ Select Master Mix (5 μL; Applied Biosystems), with 2 mM of each primer (1 μL) and 4 μL of DNA (25 ng/μL). Experiments were conducted at the Real-Time PCR Facility – RPT09D PDTIS/René Rachou Institute – FIOCRUZ/MG. The samples were incubated at 95 °C for 10 min, and then submitted to 40 cycles of 95 °C for 15 s and 60 °C for 1 min, and at each time point, fluorescence data were collected. Parasite quantification for each spleen sample was calculated by interpolation from the standard curve, performed in duplicate, and converted into number of parasites per nucleated cells (multiplied by one thousand to facilitate visualization).

### Cellular response evaluated by a capture ELISA

IFN-γ, IL-4, IL-10, IL-12p70, and GM-CSF levels were measured in the cell supernatant of spleen cells of the treated and infected animals, one and 15 days after treatment. For this, cells (5 × 10^6^ per mL) were plated in duplicate on 24-well plates (Nunc) and incubated in Dulbecco’s Modified Eagle Medium (DMEM) plus 20% FBS and 20 mM L-glutamine at pH 7.4, or stimulated with *L. infantum* SLA (50 μg/mL) for 48 h at 37 °C in 5% CO_2_. Cytokine levels were measured by a capture ELISA (BD PharMingen^®^, San Diego, CA, USA), according to the manufacturer’s instructions. Nitrite production was also evaluated in the cellular supernatant by the Griess reaction.

### Cytokine profile investigated by flow cytometry

A flow cytometry assay was performed to evaluate the IFN-γ, TNF-α and IL-10-producing CD4^+^ and CD8^+^ T cell frequency in the treated and infected animals, 15 days after treatment. For this, cells (5 × 10^6^ per mL) were incubated in complete RPMI 1640 medium in polypropylene tubes (PharMingen^®^), and were non-stimulated (medium) or stimulated with *L. infantum* SLA (50 μg/mL) for 48 h at 37 °C in 5% CO_2_. IFN-γ, TNF-α and IL-10-producing CD4^+^ and CD8^+^ T cell frequency was evaluated using an analysis based on their relative flow cytometry size (forward laser scatter – FSC) and granularity (side laser scatter – SSC) graphs. After the selection of the interest region R1 containing FSCLow and SSCLow phenotype cells, graphs of density plot distribution of CD4/FL1 or CD8/FL1 versus IFN-γ/FL2^+^, TNF-α/FL2^+^, and IL-10/FL2^+^ cells were constructed, in order to determine the IFN-γ^+^, TNF-α^+^, and IL-10^+^ T cell frequency. Results were expressed as indexes, which were calculated by the ratio between the cytokine-producing T cell percentages versus the values found in the non-stimulated culture.

### Humoral response

The anti-SLA IgG1 and IgG2a isotype levels were evaluated in sera samples of the treated and infected animals, one and 15 days after treatment. *L. infantum* SLA was used as an antigen (1.0 μg per well), and sera samples were 1:100 diluted in PBS-T (PBS 1× plus 0.05% Tween 20). Both anti-mouse IgG1 and IgG2a horseradish-peroxidase conjugated antibodies (Sigma-Aldrich, USA) were used in a 1:10,000 dilution, which was performed using PBS-T. Reactions were developed by adding H_2_O_2_, ortho*-*phenylenediamine and citrate-phosphate buffer at pH 5.0, for 30 min and in the dark. Reactions were then stopped by adding 2N H_2_SO_4_, and the optical density (OD) values were read in an ELISA microplate spectrophotometer (Molecular Devices, Spectra Max Plus, Canada) at 492 nm.

### Statistical analysis

Results were entered into Microsoft Excel (version 10.0) spreadsheets and analyzed by GraphPad Prism^TM^ (version 6.0 for Windows; GraphPad Software, La Jolla, CA, USA). The one-way analysis of variance (ANOVA) followed by Bonferroni’s post-test were used for comparisons between the groups. Differences were considered significant with *p* < 0.05. Experiments were repeated and results were similar.

## Results

### Cellular profile developed in treated and infected mice

**Figure 1:**
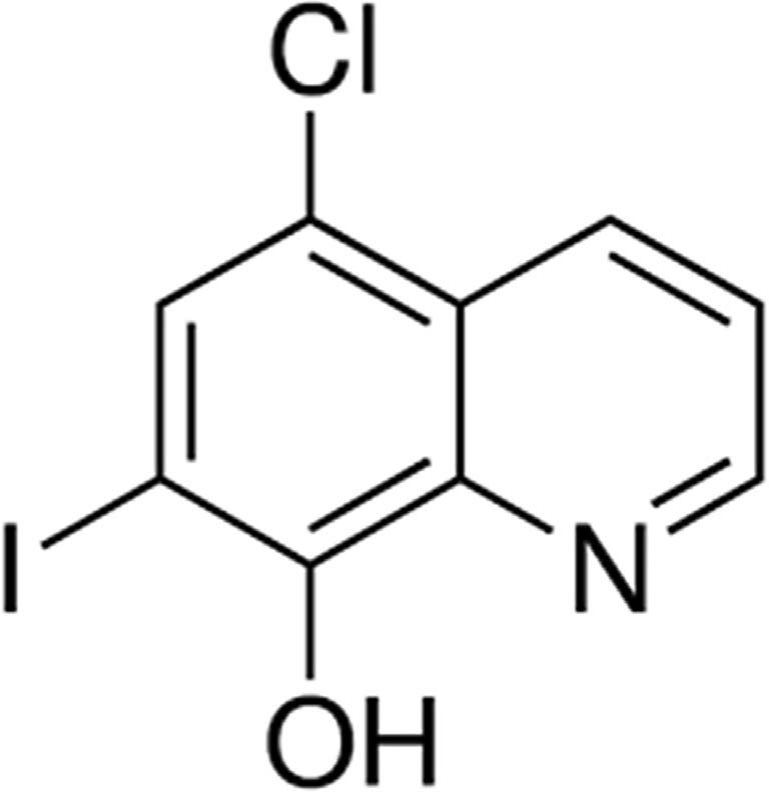
Chemical structure of the clioquinol (ICHQ) compound.

**Figure 2:**
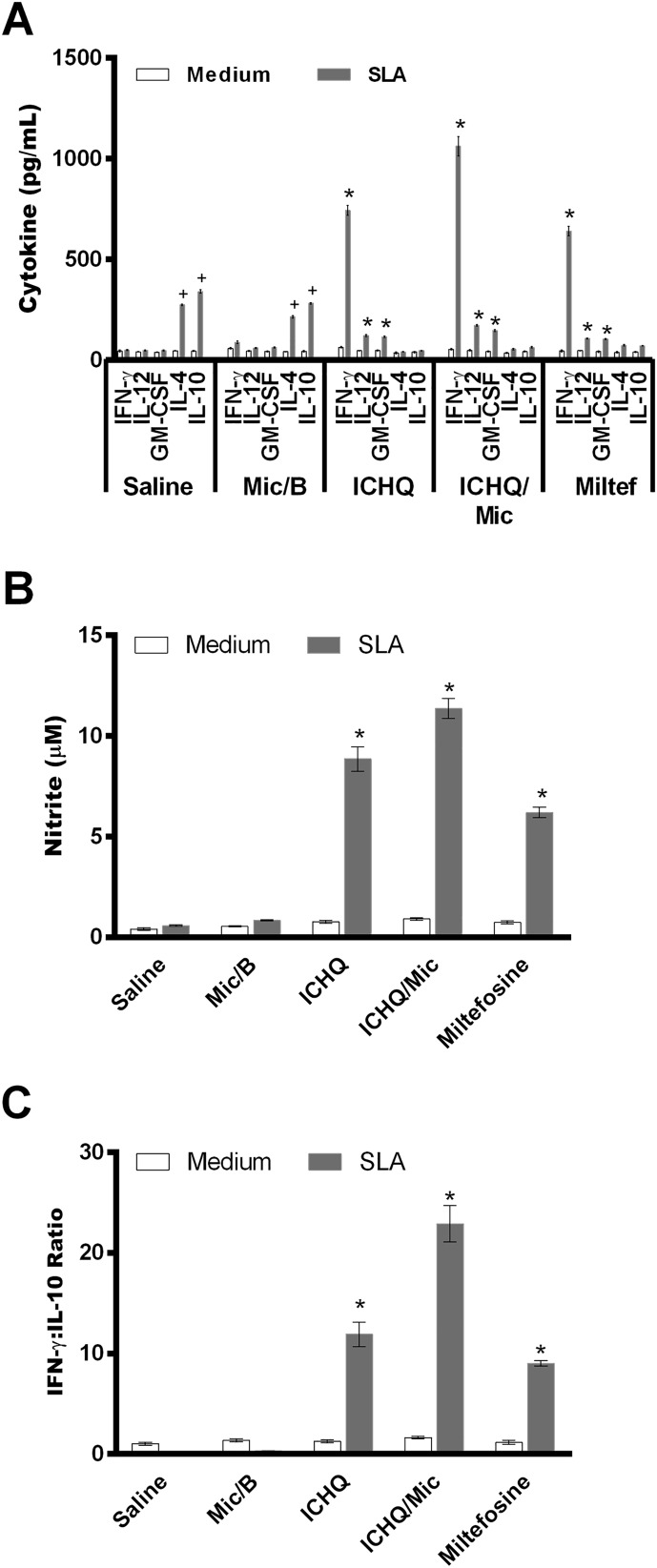
Cellular response developed in the treated and infected animals, one day after treatment. Splenocytes of the treated and infected animals were collected one day after treatment, and cells (5 × 10^6^ per mL) were non-stimulated (medium) or stimulated with *Leishmania infantum* SLA (50 μg/mL) for 48 h at 37 °C in 5% CO_2_. IFN-γ, IL-4, IL-10, IL-12p70 and GM-CSF levels were measured in the cell supernatant by a capture ELISA (A). Nitrite production was also evaluated in the cell supernatant using the Griess reaction (B). Ratios between the IFN-γ and IL-10 levels were calculated and results are shown (C). Bars represent the mean ± standard deviation of the groups. (*) indicates a statistically significant difference in relation to the saline and Mic/B groups (*p* < 0.0001). (^+^) indicates a statistically significant difference in relation to the miltefosine, ICHQ, and ICHQ/Mic groups (*p* < 0.0001).

**Figure 3:**
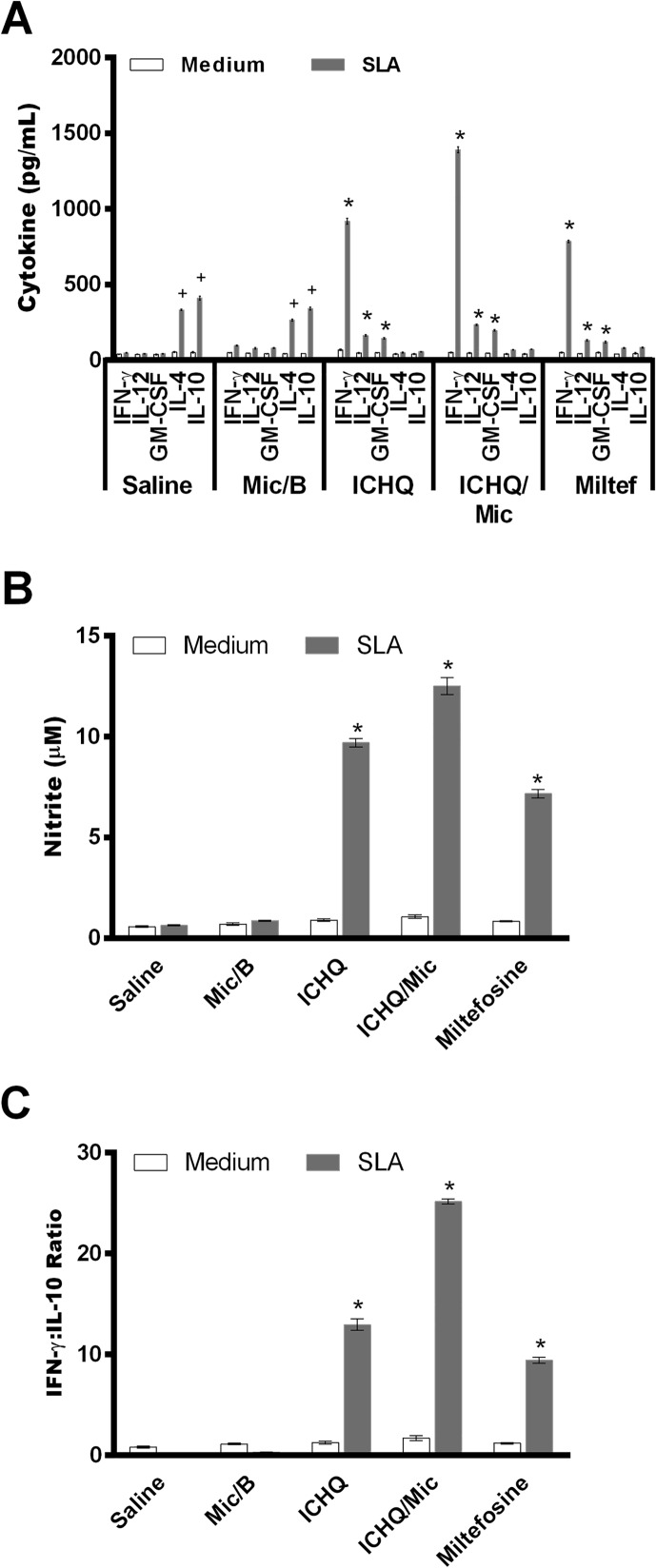
Cellular profile generated in the treated and infected animals, 15 days after treatment. Fifteen days after treatment, cellular response was also evaluated in the treated and infected animals. Spleen cells (5 × 10^6^ per mL) were non-stimulated (medium) or stimulated with *Leishmania infantum* SLA (50 μg/mL) for 48 h at 37 °C in 5% CO_2_. IFN-γ, IL-4, IL-10, IL-12p70 and GM-CSF levels were measured in the cell supernatant by a capture ELISA (A). Nitrite production was also evaluated in the cell supernatant (B). The ratios between the IFN-γ and IL-10 levels were calculated, and values are also shown (C). Bars represent the mean ± standard deviation of the groups. (*) indicates a statistically significant difference in relation to the saline and Mic/B groups (*p* < 0.001). (^+^) indicates a statistically significant difference in relation to the miltefosine, ICHQ, and ICHQ/Mic groups (*p* < 0.001).

**Figure 4:**
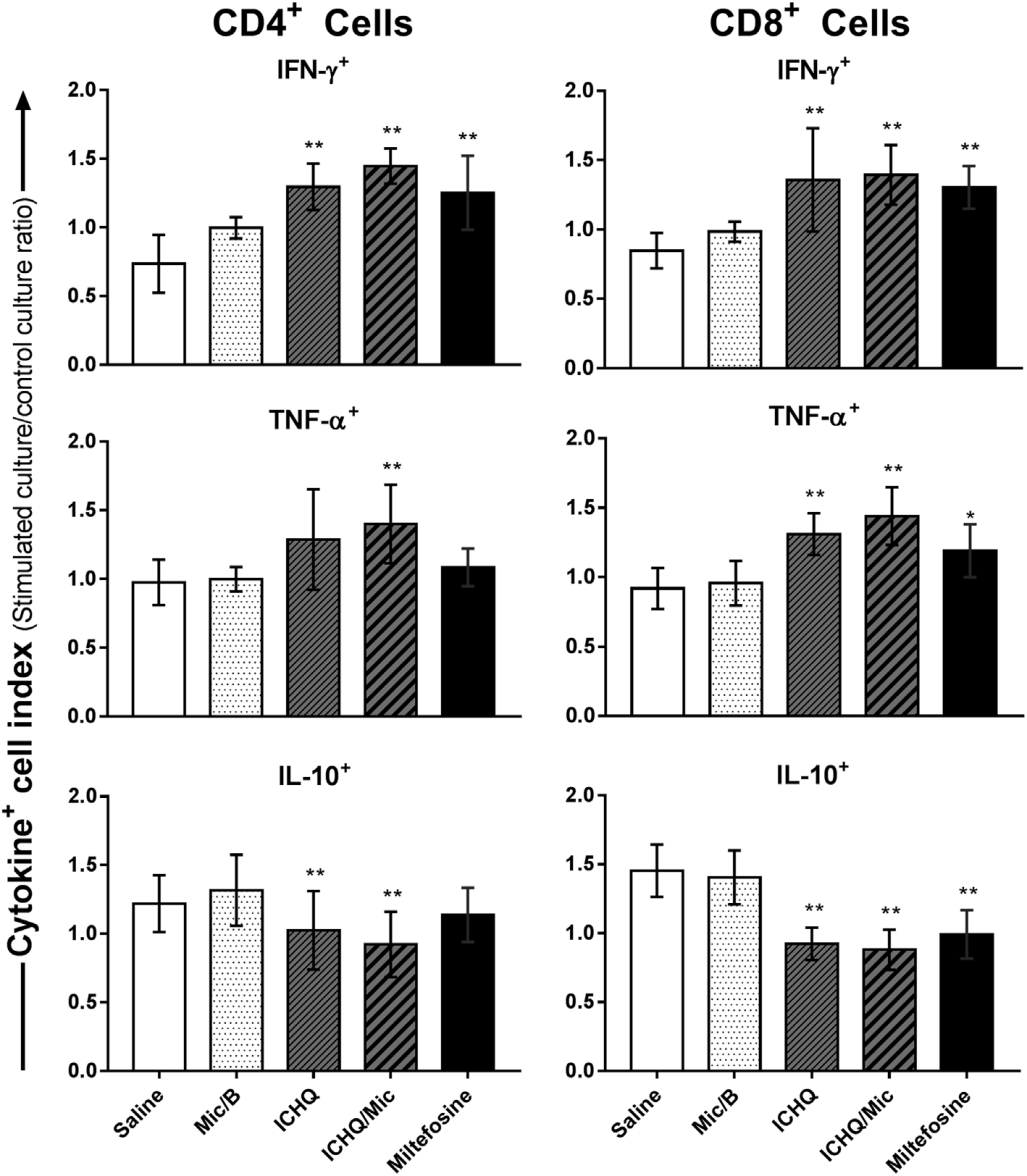
Intracytoplasmic cytokine-producing CD4^+^ and CD8^+^ T-cell frequency in the treated and infected animals. BALB/c mice were infected with *Leishmania infantum* promastigotes and received saline or were treated with empty micelle (B/Mic), miltefosine, ICHQ, or ICHQ/Mic. The animals were euthanized 15 days after treatment, when their splenocytes were collected and non-stimulated or *in vitro* stimulated with SLA (50 μg/mL) for 48 h at 37 °C in 5% CO_2_. Results were expressed as cytokine indexes (stimulated culture/control culture ratio) to obtain the IFN-γ, TNF-α and IL-10-producing CD4^+^ and CD8^+^ T cell frequency. Bars indicate the mean ± standard deviation of the groups. (*) and (**) indicate statistically significant differences in relation to the saline and B/Mic groups, respectively (*p* < 0.05).

In this study, ICHQ was incorporated in a Poloxamer 407-based micelle system, and the composition was evaluated for the treatment of *L. infantum*-infected BALB/c mice. The chemical structure of the ICHQ compound is shown in Figure 1. Initially, the cellular profile in the treated and infected animals was investigated, by means of assay of anti-parasite Th1 and Th2-type cytokines in the culture supernatant of the stimulated splenocytes. Performing the evaluations one day after treatment, results showed that spleen cells of the miltefosine, ICHQ or ICHQ/Mic-treated mice produced significantly higher IFN-γ, IL-12 and GM-CSF levels, which were associated with low IL-4 and IL-10 production. On the other hand, mice in the saline and Mic/B groups produced higher antileishmanial IL-4 and IL-10 levels (Fig. 2A). As an indicator of NO production and macrophage activation, the nitrite secretion was evaluated in the culture supernatant, and results showed that miltefosine, ICHQ or ICHQ/Mic-treated animals produced significantly higher nitrite levels, when compared to the controls (Fig. 2B). Ratios between the IFN-γ and IL-10 levels were calculated and results showed a polarized Th1-type response in these animals (Fig. 2C). Evaluating the animals 15 days after treatment, a similar cellular profile was found in the animals, since higher parasite-specific IFN-γ, IL-12, and GM-CSF levels were found, associated with low IL-4 and IL-10 levels (Fig. 3A). By contrast, spleen cells of the mice in the saline and Mic/B groups produced significantly higher IL-4 and IL-10 levels. The nitrite secretion also showed similar results to those obtained when analyses were performed one day after treatment, indicating persistent macrophage activation to kill internalized parasites (Fig. 3B). The ratios between IFN-γ and IL-10 levels were also calculated and results showed the maintenance of Th1-type profiles in these animals (Fig. 3C). A flow cytometry assay indicated that the treatment with miltefosine, ICHQ or ICHQ/Mic induced higher IFN-γ and TNF-α-producing CD4^+^ and CD8^+^ T-cell frequency, when compared to the controls (Fig. 4). Comparing the results between the treatments, the ICHQ/Mic composition presented higher Th1-type T cell frequency, when compared to values found in the miltefosine or ICHQ treated groups.

### Humoral response generated in the treated and infected animals

**Figure 5:**
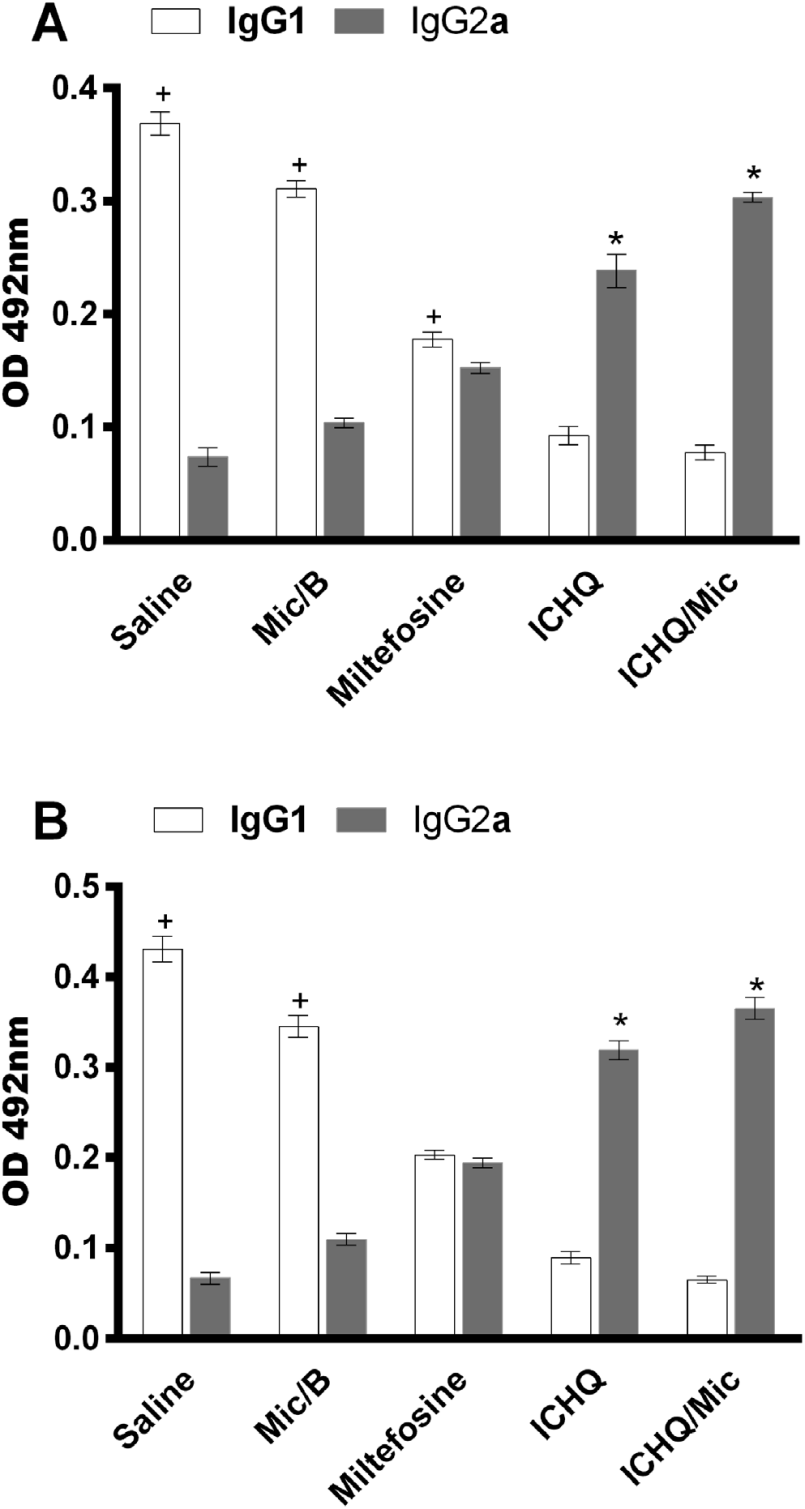
Humoral response developed in the treated and infected animals. Serum samples were collected from the treated and infected animals, one (A) and 15 (B) days after treatment, when the anti-parasite IgG1 and IgG2a isotype levels were evaluated. White and grey bars indicate the mean ± standard deviation of the IgG1 and IgG2a levels, respectively. (*) indicates a statistically significant difference in relation to the saline and Mic/B groups (*p* < 0.001). (^+^) indicates a statistically significant difference in relation to the miltefosine, ICHQ, and ICHQ/Mic groups (*p* < 0.001).

Anti-parasite IgG1 and IgG2a isotype antibody levels were evaluated one and 15 days after treatment (Fig. 5). Evaluating the samples one day after treatment, results showed that miltefosine, ICHQ or ICHQ/Mic-treated mice produced higher levels of antileishmanial IgG2a isotype than IgG1 isotype. However, IgG1 production was higher in the control (saline and Mic/B) groups. Results obtained 15 days after treatment indicated maintenance of the humoral profile in the animals, and those treated with ICHQ/Mic presented the most polarized anti-parasite IgG2a-based humoral response, when compared to the values found in the other groups. Again, parasite-specific IgG1 production was higher in the saline and Mic/B groups.

### Parasitological evaluation in the treated and infected mice

**Figure 6:**
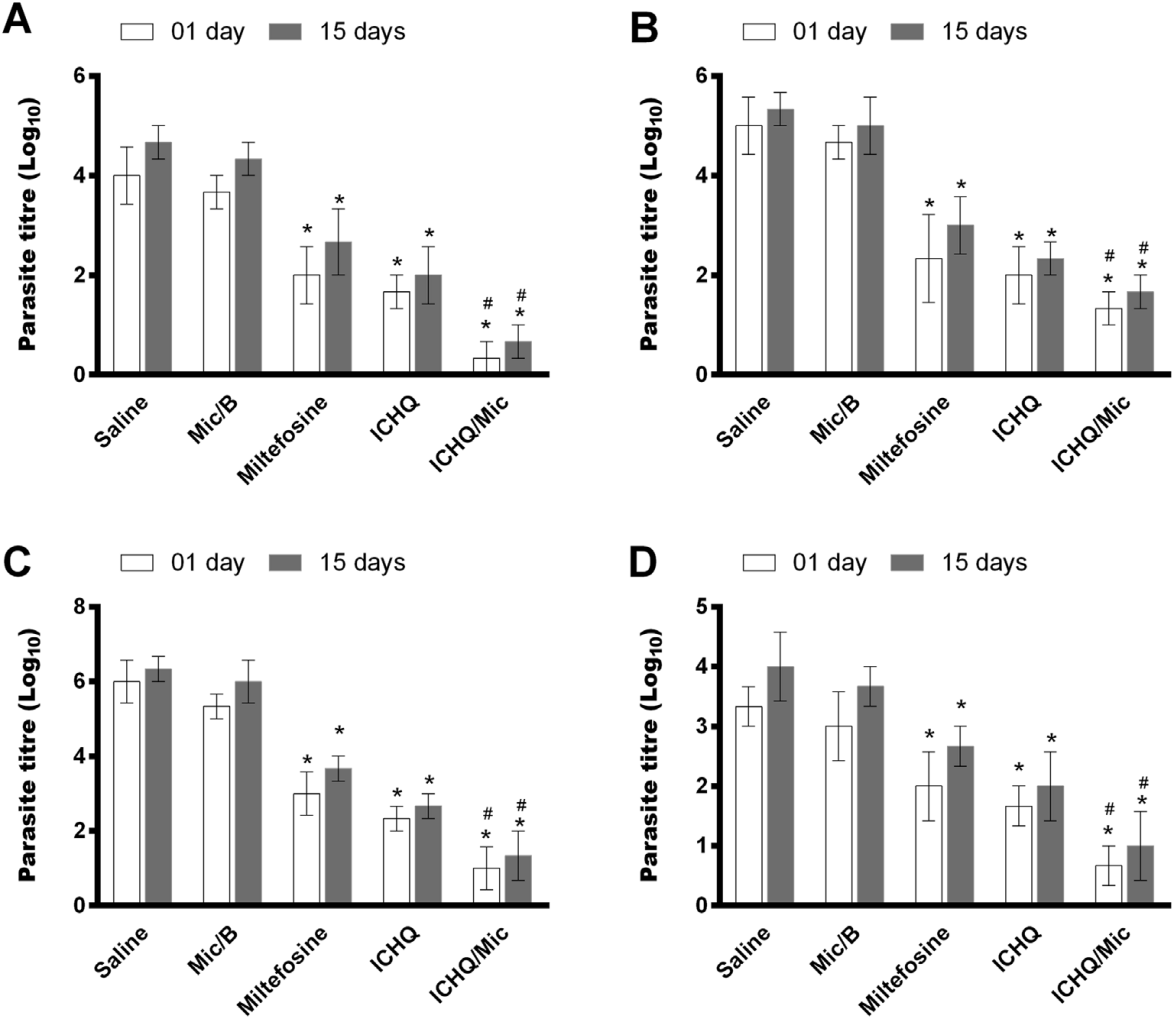
Parasite load evaluated by a limiting dilution assay. To evaluate the *in vivo* antileishmanial action of ICHQ incorporated or not in a micellar system, BALB/c mice (*n* = 12 per group) were subcutaneously infected with 10^7^ *Leishmania infantum* stationary promastigotes. 45 days after infection, they received subcutaneous injections every 2 days for 10 days with: saline, empty micelle (Mic/B), ICHQ, ICHQ/Mic, or miltefosine (oral route). Half of the animals per group were euthanized one and 15 days after treatment, when liver, spleen, bone marrow and draining lymph nodes were collected for evaluation of the parasite load. White and grey bars indicate the mean ± standard deviation of the groups, one and 15 days after treatment, respectively, in liver (A), spleen (B), bone marrow (C), and draining lymph nodes (D). (*) indicates a statistically significant difference in relation to the saline and Mic/B groups (*p* < 0.001). (^#^) indicates a statistically significant difference in relation to the miltefosine and ICHQ groups (*p* < 0.01).

**Figure 7:**
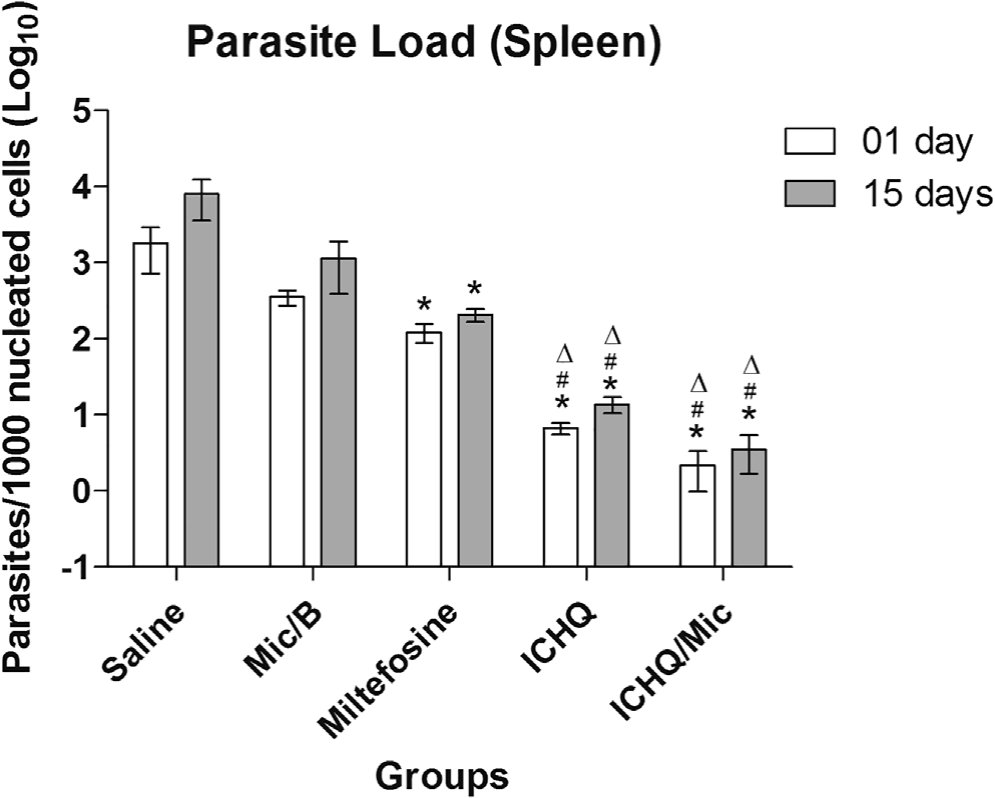
Splenic parasitism evaluated by RT-PCR. Splenic parasitism was also evaluated by RT-PCR, one and 15 days after treatment. Results were converted into number of parasites (in log) per nucleated cell (multiplied by one thousand to facilitate visualization). Bars indicate the mean ± standard deviation of the groups. (*), (^#^) and (^Δ^) indicate statistically significant differences in comparison to the saline, Mic/B and miltefosine groups, respectively (*p* < 0.05).

*In vivo* antileishmanial activity was evaluated by means of the evaluation of parasite load in distinct organs of the treated and infected mice, one and 15 days after treatment. Results showed that the treatment using miltefosine, ICHQ or ICHQ/Mic induced to most significant reductions in the parasite load in liver, spleen, BM and dLNs of the animals, when compared to the controls, in both periods of time after treatment (Fig. 6). A comparison performed between the treatments suggested that the ICHQ/Mic composition induced to the highest reductions in the parasite burden, when compared to the other groups. Similar results were obtained when the splenic parasite load was evaluated by RT-PCR (Fig. 7). In addition, no organic toxicity was found when ICHQ and ICHQ/Mic were used in the animals.

## Discussion

Visceral leishmaniasis is a neglected tropical disease in the world, which affects mainly poorer populations in developing countries [61]. Current treatment is based on the use of pentavalent antimonials; however, they can cause severe side effects in patients, such as hepatic, cardiac and renal toxicity [53]. In addition, less toxic drugs are more expensive or have limited availability. In this context, the identification of new antileishmanial agents should be performed. Interest in the use of natural products to treat diseases such as leishmaniasis has increased in recent decades. For instance, naphthoquinones and quinolines have been used in *in vitro* experiments against various *Leishmania* spp., and positive results have been obtained [2, 7]. These compounds are natural aromatic metabolites found in several plant families, and they present relevant biological activity, such as antileishmanial action [37, 46].

In a recent study, our research group identified a quinoline-derived molecule called clioquinol or ICHQ, which was tested *in vitro* against *L. amazonensis* and *L. infantum*. Results showed significant antileishmanial activity against both parasite species, in addition to low toxicity in murine and human cells and efficacy in treating infected macrophages [55]. In addition, ICHQ was incorporated in a Poloxamer 407-based polymeric system, and the composition showed *in vivo* efficacy against *L. amazonensis* infection in BALB/c mice, since significant reductions in the average lesion diameter and parasite burden were found, along with the development of an antileishmanial Th1-type response [56]. With the aim of identifying new antileishmanial agents, in the present study, ICHQ/Mic was evaluated against *L. infantum* infection.

Results obtained demonstrated that the treatment of the infected mice induced significant reductions in the parasite load in all evaluated organs, and animals developed anti-parasite Th1-type immunity, which was based on the production of high levels of IFN-γ, IL-12 and GM-CSF, associated with low IL-4 and IL-10 production, with predominance of anti-SLA IgG2a isotype antibody. More importantly, parasitological and immunological correlates were similar when two different endpoints were evaluated, one and 15 days after treatment, suggesting possible long-term activity of the composition against *L. infantum* infection. This is important mainly because studies usually evaluate one endpoint after antileishmanial therapy [6, 13, 42].

Miltefosine is a drug used to treat leishmaniasis [22, 58]. It has been shown to be effective against various parasite species, such as those causing visceral leishmaniasis and/or tegumentary leishmaniasis [8, 19, 44]. However, recent reports have shown that miltefosine can cause adverse effects such as nausea and vomiting during and/or after administration as well as teratogenicity. As a consequence, the compound is contra-indicated in pregnancy [51]. In addition, disease relapse and the occurrence of post kala-azar dermal leishmaniasis after treatment have been reported [14].

In the present study, we used ICHQ/Mic, which was recently suggested to be effective against murine TL [56], to treat *L. infantum*-infected BALB/c mice, a parasite species responsible for VL cases in the Americas. Results showed that the composition was effective in reducing the parasite load in the treated and infected animals, and stimulated the development of Th1-type immune response in such animals, when two distinct endpoints were evaluated. Although we did not perform a full dose-response study comparing the efficacy between ICHQ/Mic and miltefosine, and this is a limitation of our work, our data suggest that ICHQ/Mic could be, at least, considered for future studies as a therapeutic agent for the treatment of VL.

The development of specific Th1-type immunity is a requirement for *Leishmania* control in infected hosts [48]. In this context, the production of cytokines, such as IFN-γ and IL-12, among other pro-inflammatory molecules, is considered detrimental to stimulate infected cells to kill internalized parasites [33, 64]. Here, the treatment of the infected mice using miltefosine also induced the activation of Th1-type immune cells, with high levels of antileishmanial IFN-γ, IL-12 and GM-CSF cytokines being found in the cell supernatant of the stimulated cells. These findings are consistent with reports in the literature on the protective effect of miltefosine to treat *Leishmania* infection [38]. ICHQ/Mic-treated mice also presented a Th1-type immune response and, more importantly, they did not show adverse effects and/or inflammatory reactions after the administration of doses. This suggests the absence of toxicity caused by this composition in the animals, as described in other studies [48, 55, 56]. Considering that infected macrophages, when activated by Th1-type cytokines, can kill *Leishmania* parasites [57], we measured the nitrite levels in the treated and infected animals. Based on the finding that spleen cells of ICHQ/Mic-treated and infected mice responded with higher IFN-γ production after re-stimulation with the parasite protein extract, we suggest a correlation between the cellular response developed in the animals and the reduced parasitism found in the evaluated organs.

Quinoline-derivative molecules have shown antileishmanial activity in both *in vitro* and/or *in vivo* experiments against different *Leishmania* spp., such as *L. amazonensis, L. mexicana*, and *L. donovani*, among others [11, 17, 24]. However, when used to treat infected mice, high daily doses (between 10 and 50 mg per kg body weight) are usually administered to the animals [32, 42, 47]. Here, we reported the *in vivo* efficacy of ICHQ/Mic against *L. infantum* infection by using 5.0 mg of ICHQ per kg body weight. We administered the composition for 10 days, with a two-day interval, via the subcutaneous route. In this context, only five doses were administered. In other studies, higher numbers of doses and concentrations of antileishmanial compounds are usually employed in the animals, aiming to enhance their efficacy [21, 36, 42]. The results found here indicated a 90% reduction in the parasite load in the treated and infected animals, when compared to the other groups. In addition, the hepatic parasite load was almost completely eliminated, with a 95.0% reduction being found in the ICHQ/Mic-treated animals. This suggests a positive systemic effect of this composition against VL.

Poloxamer 407 (Pluronic F127) is a non-ionic surfactant formed by symmetric tri-block copolymers composed of propylene oxide and ethylene oxide [26]. It has a hydrophobic core and a hydrophilic shell, which can harbor the amphiphilic moiety and prevent the direct exposure to vital organs [29]. The interface formed by the hydrophilic block prevents micellar aggregation, protein recognition, and non-specific adherence, thereby sparing the body from the adverse effects induced by antileishmanial compounds [47]. Poloxamer 407-based formulations have been successfully tested against leishmaniasis, and they present advantages when compared to traditional formulations, such as efficacy, target orientation, low toxicity, and low cost [27]. These formulations have been administered by the subcutaneous route in murine models, since a semi-rigid gel is formed in contact with the local tissue, creating a reservoir system to maintain the product in the extracellular space. In the course of hours, the gel matrix is diluted by body fluids and the product is gradually released into the circulation, enabling its systemic action in a controlled manner [3]. This allows administration of the formulations with a higher time interval between doses, and makes it possible to reduce the number of applications and/or treatment times [18, 34, 35, 56].

The absence of other treatment regimens, such as the use of other antileishmanial products to compare with data obtained using miltefosine and ICHQ/Mic, as well as the absence of parasitological and immunological evaluations performed over longer periods of time after treatment are considered limitations of this study. Nevertheless, our data suggest that ICHQ/Mic presents *in vivo* antileishmanial activity against *L. infantum*, and could be tested in future studies as a candidate for the treatment of visceral leishmaniasis.

## Conflict of interest

The authors confirm that they have no conflicts of interest in relation to this work.

## Notes

**Cite this article as**: Tavares GSV, Mendonça DVC, Pereira IAG, Oliveira-da-Silva JA, Ramos FF, Lage DP, Machado AS, Carvalho LM, Reis TAR, Perin L, Carvalho AMRS, Ottoni FM, Ludolf F, Freitas CS, Bandeira RS, Silva AM, Chávez-Fumagalli MA, Duarte MC, Menezes-Souza D, Alves RJ, Roatt BM & Coelho EAF. 2020. A clioquinol-containing Pluronic^®^ F127 polymeric micelle system is effective in the treatment of visceral leishmaniasis in a murine model. Parasite **27**, 29.
